# Phosphate clearance in peritoneal dialysis

**DOI:** 10.1038/s41598-020-74412-2

**Published:** 2020-10-15

**Authors:** Malgorzata Debowska, Rafael Gomez, Joyce Pinto, Jacek Waniewski, Bengt Lindholm

**Affiliations:** 1 Department of Mathematical Modeling of Physiological Processes, Nalecz Institute of Biocybernetics and Biomedical Engineering, Polish Academy of Sciences, Warsaw, Poland; 2 RTS SAS, Cali, Colombia; 3 Renal Medicine and Baxter Novum, Department of Clinical Science, Intervention and Technology, Karolinska Institutet, Stockholm, Sweden

**Keywords:** Peritoneal dialysis, End-stage renal disease

## Abstract

In renal failure, hyperphosphatemia is common and correlates with increased mortality making phosphate removal a key priority for dialysis therapy. We investigated phosphate clearance, removal and serum level, and factors associated with phosphate control in patients undergoing continuous ambulatory (CAPD), continuous cyclic (CCPD) and automated (APD) peritoneal dialysis (PD). In 154 prevalent PD patients (mean age 53.2 ± 17.6 year, 59% men, 47% anuric), 196 daily collections of urine and 368 collections of dialysate were evaluated in terms of renal, peritoneal and total (renal plus peritoneal) phosphorus removal (g/week), phosphate and creatinine clearances (L/week) and urea KT/V. Dialytic removal of phosphorus was lower in APD (1.34 ± 0.62 g/week) than in CAPD (1.89 ± 0.73 g/week) and CCPD (1.91 ± 0.63 g/week) patients; concomitantly, serum phosphorus was higher in APD than in CAPD (5.55 ± 1.61 vs. 4.84 ± 1.23 mg/dL; *p* < 0.05). Peritoneal and total phosphate clearances correlated with peritoneal (rho = 0.93) and total (rho = 0.85) creatinine clearances (*p* < 0.001) but less with peritoneal and total urea KT/V (rho = 0.60 and rho = 0.65, respectively, *p* < 0.001). Phosphate removal, clearance and serum levels differed between PD modalities. CAPD was associated with higher peritoneal removal and lower serum level of phosphate than APD.

## Introduction

Phosphate retention is a major problem in patients with chronic kidney disease (CKD)^1^. The dialytic removal of phosphorus combined with use of phosphate binders are usually inadequate to counteract the intestinal absorption of phosphorus in patients with renal failure^2^. This leads to hyperphosphatemia and secondary hyperparathyroidism, which are associated with adverse cardiovascular outcomes and contribute to increased risk of death^1,3–6^. Therefore, a better understanding of phosphorus removal by dialysis is of high importance in CKD patients.

In this study, we investigated associations of weekly renal, peritoneal and total (renal plus peritoneal) phosphate clearance, removal and serum phosphorus concentration with different parameters of dialysis and patient characteristics in patients treated by continuous ambulatory (CAPD), continuous cyclic (CCPD) and automated (APD) peritoneal dialysis (PD).

## Methods

### Ethics statement

The research was conducted in accordance with the Declaration of Helsinki as part of the routine clinical evaluation. All procedures performed in the study, involving patients, were in accordance with the ethical standards required by the institutional and national research committees for the time of study duration. Approval was granted by the RTS Ethical and Investigation Committee (September 2016). The informed consent was not required but each patient gave informed consent to perform laboratory measurements and for data management.

### Patients and study design

This observational, cross-sectional study included 154 prevalent PD patients (at the dialysis facilities of RTS Versalles, Cali, Colombia) who were investigated as part of their routine clinical evaluation. In each patient at least one daily collection of dialysate and urine (if non-anuric) was carried out. In total, 196 daily collections of urine and 368 collections of dialysate (from 1 to 8 measurements for each patient) were evaluated. CAPD was performed in 48 patients (120 measurements), CCPD (with wet day; meaning presence of dialysis fluid in the abdominal cavity) in 25 patients (61 measurements) and APD (with dry day; dialysis fluid was drained at the end of APD session) in 81 patients (187 measurements), (Table 1). During study duration patients were instructed by dietitian not to take more than 800 mg of phosphate per day, but the exact amount of phosphate intake was not assessed. Phosphate binders were prescribed to all patients with the dose regulated according to plasma phosphate. This study is an extension of our previous analysis^7^ performed in 73 patients undergoing similar measurement protocol and comprising 20% of measurements analyzed in this study.

**Table 1:** Demographic and laboratory characteristics of patients on continuous ambulatory (CAPD), continuous cyclic (CCPD), automated peritoneal dialysis (APD) and for all pooled therapies.

	(1) CAPD	(2) CCPD	(3) APD	All
Number of patients	48 (31%)	25 (16%)	81 (53%)	154
Number of measurements	120 (33%)	61 (17%)	187 (51%)	368
Transport type, 1/2/3/4^(a)^	19%/38%/37%/5%	18%/30%/44%/8%	17%/30%/32%/17%	18%/33%/36%/11%
Creatinine PET D/P	0.61 ± 0.13	0.62 ± 0.12	0.64 ± 0.13	0.63 ± 0.13
Anuric^(^***^)^	37%^(2)^	84%^(1,3)^	41%^(2)^	47%
Urine volume, mL/day^(^***^)^	427.54 ± 472.38^(2)^	59.43 ± 149.96^(1,3)^	381.43 ± 492.54^(2)^	343.09 ± 464.15
Gender, male^(^***^)^	44%^(2)^	80%^(1,3)^	53%^(2)^	59%
Age, year^(^***^)^	47.63 ± 17.64^(3)^	47.92 ± 16.44^(3)^	58.51 ± 16.28^(1,2)^	53.21 ± 17.56
Weight, kg	65.30 ± 14.60	68.78 ± 11.93	64.87 ± 13.41	65.66 ± 13.62
Height, m^(^***^)^	1.62 ± 0.09^(2)^	1.68 ± 0.07^(1,3)^	1.62 ± 0.09^(2)^	1.63 ± 0.09
Body mass index, kg/m^2^	24.52 ± 3.99	24.58 ± 4.47	24.79 ± 4.72	24.66 ± 4.44
Body surface area, m^2^^(^**^)^	1.69 ± 0.22^(2)^	1.77 ± 0.15^(1,3)^	1.68 ± 0.18^(2)^	1.70 ± 0.19
Total body water, L^(^***^)^	35.51 ± 6.43^(2)^	39.22 ± 4.41^(1,3)^	34.95 ± 6.17^(2)^	35.84 ± 6.18
Serum creatinine, mg/dL^(^***^)^	11.72 ± 4.46^(2)^	14.63 ± 3.57^(1,3)^	11.08 ± 4.00^(2)^	11.88 ± 4.27
Serum urea, mg/dL	47.85 ± 14.41	45.49 ± 14.02	45.38 ± 12.37	46.20 ± 13.35
Serum phosphorus, mg/dL^(^***^)^	4.84 ± 1.23^(3)^	5.21 ± 1.40	5.55 ± 1.61^(1)^	5.26 ± 1.49
Serum glucose, mg/dL	122.00 ± 70.61	117.77 ± 35.07	121.76 ± 65.78	121.18 ± 63.35
Serum albumin, mg/dL^(^***^)^	3.73 ± 0.50^(2)^	4.10 ± 0.64^(1,3)^	3.62 ± 0.50^(2)^	3.73 ± 0.55

^(a)^1, 2, 3, 4, denote slow, slow average, fast average and fast transport types, respectively; in 1% in CAPD, 4% in APD and in total in 2% of measurements the transport type was not evaluated.

^(^***^)^ and ^(^**^)^ denote global *p* value < 0.001 and < 0.01, respectively, during multicomparison procedure.

^(1,2,3)^Superscripts mean significant difference (*p* < 0.05) between the current value and the therapy listed in bracket.

All bags with drained dialysate were brought by CAPD patients to the clinic for volume measurement, while in APD and CCPD patients, dialysis drainage volumes and ultrafiltration volumes were derived from cycler (HomeChoice, Baxter Healthcare, Deerfield, Illinois, USA). Phosphate, urea and creatinine concentrations were measured in collections of dialysate and urine, and in serum. Urea and creatinine were assayed by routine methods. Phosphorus concentration was determined using direct UV measurement of phosphomolybdate complex. Peritoneal transport type was evaluated by peritoneal equilibration test (PET).

### Calculation of renal, peritoneal and total clearances

Weekly renal clearance was evaluated for phosphate and creatinine from 24-h collection of urine:

1 $${\text{Weekly}}\,{\text{renal}}\,{\text{clearance}} = 7\frac{{{\text{C}}_{{{\text{urine}}}} \cdot {\text{V}}_{{{\text{urine}}}} }}{{{\text{C}}_{{{\text{serum}}}} }} \cdot \frac{1}{{1\,{\text{week}}}} \cdot \frac{1.73}{{{\text{BSA}}}}\,[{\text{L/week}}],$$

where C_urine_—solute concentration in urine, V_urine_—urine volume, C_serum_—solute concentration in blood serum and BSA—body surface area. Solute mass removed by the kidneys is equal to C_urine_·V_urine_. Weekly peritoneal clearances for phosphate and creatinine were calculated based on measurements in 24-h collection of drained dialysate:

2 $${\text{Weekly}}\,{\text{peritoneal}}\,{\text{clearance}} = 7\frac{{{\text{C}}_{{{\text{dialysate}}}} \cdot {\text{V}}_{{{\text{dialysate}}}} }}{{{\text{C}}_{{{\text{serum}}}} }} \cdot \frac{1}{{1\,{\text{week}}}} \cdot \frac{1.73}{{{\text{BSA}}}}\,[{\text{L/week}}],$$

where C_dialysate_—solute concentration in drained dialysate and V_dialysate_—drainage volume. C_dialysate_·V_dialysate_ is the mass of solute removed by dialysis. Total weekly clearance was determined as the sum of renal and peritoneal clearances:

3 $${\text{Total}}\,{\text{weekly}}\,{\text{clearance}} = {\text{Weekly}}\,{\text{renal}}\,{\text{clearance}} + {\text{weekly}}\,{\text{peritoneal}}\,{\text{clearance}}\,[{\text{L/week}}].$$

In anuric patients, the total weekly clearance is equal to peritoneal clearance.

Urea KT/V was estimated based on 24-h collections of urine and drained dialysate:

4 $${\text{Weekly}}\,{\text{renal}}\,{\text{urea}}\frac{{{\text{KT}}}}{{\text{V}}} = 7\frac{{{\text{C}}_{{{\text{urine}}}} \cdot {\text{V}}_{{{\text{urine}}}} }}{{{\text{C}}_{{{\text{serum}}}} \cdot {\text{TBW}}}},$$

5 $${\text{Weekly}}\,{\text{peritoneal}}\,{\text{urea}}\frac{{{\text{KT}}}}{{\text{V}}} = 7\frac{{{\text{C}}_{{{\text{dialysate}}}} \cdot {\text{V}}_{{{\text{dialysate}}}} }}{{{\text{C}}_{{{\text{serum}}}} \cdot {\text{TBW}}}},$$

where TBW—total body water calculated using Watson formula^8^. Total urea KT/V was determined by adding renal and peritoneal urea KT/V.

### Statistical analysis

Data are expressed as mean with ± 1 standard deviation (SD) or as number and percentage. Statistical significance was set at *p* value < 0.05, unless otherwise indicated. Multiple comparisons were investigated by Kruskal–Wallis test followed by multiple pairwise comparison analysis based on Scheffé's method. Chi-squared test was used to compare categorical variables. The relationship between two variables was tested using *weighted* Spearman correlation. In multivariate regression analysis, the mixed-model methodology was applied. The *stepwise* approach was applied for variable addition or removal to maximize total explained variance (R^2^) in multivariate regression models to examine dependencies between clearance, serum concentration and removal of phosphate and various combinations of other data. Statistical analyses were performed in Matlab R2019b (MathWorks, Natick, MA, USA) and R ver. 3.5.3.

## Results

### Characteristics of patients and therapies

In the investigated therapies (CAPD, CCPD and APD), peritoneal transport types were similarly distributed with 17–19%, 30–38%, 32–44% and 5–17% measurements belonging to slow, slow-average, fast-average and fast transport types, respectively (Table 1). In CCPD, there were significantly more anuric and more male patients (Table 1). APD patients were on average older than CAPD and CCPD patients (Table 1). CCPD patients were taller, had larger body surface area and higher volume of total water than patients on CAPD and APD (Table 1). The levels of serum creatinine and albumin were higher in patients on CCPD than in CAPD and APD patients (Table 1). Serum phosphorus was higher in APD than in CAPD patients (Table 1).

Dialysis time per day was significantly shorter in APD (10.03 ± 0.43 h/day) than in CCPD (21.51 ± 4.15 h/day) and CAPD (23.43 ± 2.54 h/day) patients (Table 2). The number of cycles also differed, ranging from 3.88 ± 0.44 (CAPD) to 5.87 ± 0.67 (CCPD) exchanges/day (Table 2). Daily infused and drained volumes of dialysis fluid differed as well with the average infused volume being 8.19 ± 1.58, 12.60 ± 1.60 and 10.20 ± 1.52 L and average drained volume 9.62 ± 1.74, 14.20 ± 1.76 and 11.39 ± 1.71 L in CAPD, CCPD and APD patients, respectively (Table 2).

**Table 2:** Solute transport data from 24-h collection of dialysate and urine in patients on continuous ambulatory (CAPD), continuous cyclic (CCPD), automated peritoneal dialysis (APD) and for all pooled therapies.

	(1) CAPD n = 48	(2) CCPD n = 25	(3) APD n = 81	All n = 154
Dialysis time, h/day^(^***^)^	23.43 ± 2.54^(3)^	21.51 ± 4.15^(3)^	10.03 ± 0.43^(1,2)^	16.30 ± 6.79
Cycle, no/day^(^***^)^	3.88 ± 0.44^(2,3)^	5.87 ± 0.67^(1,3)^	4.82 ± 0.62^(1,2)^	4.69 ± 0.88
Infused volume, L/day^(^***^)^	8.19 ± 1.58^(2,3)^	12.60 ± 1.60^(1,3)^	10.20 ± 1.52^(1,2)^	9.94 ± 2.15
Drained volume, L/day^(^***^)^	9.62 ± 1.74^(2,3)^	14.20 ± 1.76^(1,3)^	11.39 ± 1.71^(1,2)^	11.28 ± 2.30
Ultrafiltration, L/day^(^***^)^	1.42 ± 0.50^(3)^	1.60 ± 0.69^(3)^	1.19 ± 0.44^(1,2)^	1.33 ± 0.53
**Weekly urea KT/V**				
Peritoneal urea KT/V^(^***^)^	1.72 ± 0.36^(3)^	1.85 ± 0.26^(3)^	1.59 ± 0.33^(1,2)^	1.68 ± 0.35
Renal urea KT/V^(^***^)^	0.34 ± 0.43^(2)^	0.04 ± 0.10^(1,3)^	0.42 ± 0.54^(2)^	0.33 ± 0.48
Total urea KT/V^(^*^)^	2.06 ± 0.39^(2)^	1.89 ± 0.27^(1)^	2.00 ± 0.55	2.01 ± 0.47
**Weekly clearance, L/week**				
Peritoneal creatinine clearance^(^***^)^	48.43 ± 13.83^(3)^	47.39 ± 10.11^(3)^	34.36 ± 10.79^(1,2^)	41.11 ± 13.60
Renal creatinine clearance^(^***^)^	24.69 ± 37.58^(2)^	2.95 ± 7.82^(1,3)^	30.03 ± 44.03^(2)^	23.80 ± 39.28
Total creatinine clearance^(^***^)^	73.12 ± 35.11^(2,3)^	50.34 ± 12.22^(1)^	64.39 ± 42.06^(1)^	64.91 ± 37.12
Peritoneal phosphate clearance^(^***^, a)^	41.50 ± 14.50^(3)^	36.68 ± 9.56^(3)^	25.42 ± 10.18^(1,2)^	32.53 ± 13.81
Renal phosphate clearance^(^***^, a)^	10.98 ± 14.13^(2)^	1.86 ± 7.63^(1,3)^	13.80 ± 18.40^(2)^	10.90 ± 16.24
Total phosphate clearance^(^***^, a)^	52.49 ± 16.42^(2,3)^	38.54 ± 12.34^(1)^	39.23 ± 19.63^(1)^	43.44 ± 18.64
**Weekly phosphorus removal, g/week**				
Peritoneal phosphorus removal^(^***^)^	1.89 ± 0.73^(3)^	1.91 ± 0.63^(3)^	1.34 ± 0.62^(1,2)^	1.61 ± 0.72
Renal phosphorus removal^(^***^)^	0.55 ± 0.75^(2)^	0.11 ± 0.48^(1,3)^	0.67 ± 0.86^(2)^	0.54 ± 0.80
Total phosphorus removal^(^***^)^	2.44 ± 0.94^(2,3)^	2.02 ± 0.85^(1)^	2.01 ± 1.00^(1)^	2.15 ± 0.97

^(^***^)^ and ^(^*^)^ denote *p* value < 0.001 and < 0.05 during multi-comparison procedure.

^(a)^Means significant difference versus creatinine clearance in all investigated therapies and for pooled data.

^(1,2,3)^Superscripts mean significant difference (*p* < 0.05) between the current value and the therapies listed in bracket.

### Weekly urea KT/V and creatinine clearance

Peritoneal urea KT/V was lower in APD (1.59 ± 0.33) than in CAPD (1.72 ± 0.36) and CCPD (1.85 ± 0.26). Renal urea KT/V in CCPD patients was close to zero due to the fact that 84% of CCPD patients were anuric (Tables 1, 2). Total urea KT/V was higher in CAPD than in CCPD (2.06 ± 0.39 vs. 1.89 ± 0.27), (Table 2).

Peritoneal creatinine clearance was significantly lower in APD (34.36 ± 10.79 L/week) than in CAPD patients (48.43 ± 13.83 L/week) despite similar percentage of anuric patients in both therapies (41% and 37% in APD and CAPD, respectively), (Tables 1, 2). Renal creatinine clearance in CCPD patients was small (Table 2). Total creatinine clearance was the highest in CAPD (73.12 ± 35.11 L/week) and lower in APD (64.39 ± 42.06 L/week) and CCPD (50.34 ± 12.22 L/week) patients (Table 2).

### Weekly clearance and removal of phosphorus

Phosphate clearance (peritoneal, renal and total) was significantly lower than creatinine clearance (Table 2). Peritoneal phosphate clearance was significantly lower in APD than in CCPD and CAPD patients: 25.42 ± 10.18 versus 36.68 ± 9.56 and 41.50 ± 14.50 L/week, respectively (Table 2). Renal phosphate clearance was similar in CAPD and APD patients and very small in CCPD patients (Table 2). Total phosphate clearance was the highest in CAPD (52.49 ± 16.42 L/week), followed by APD (39.23 ± 19.63 L/week) and CCPD (38.54 ± 12.34 L/week), (Table 2). The pattern of values was similar for phosphate and creatinine clearances (peritoneal, renal and total) in the studied therapies with peritoneal clearance of phosphate and creatinine being the lowest in APD patients (Table 2).

Dialytic removal of phosphorus was lower in APD patients (1.34 ± 0.62 g/week) than in CAPD (1.89 ± 0.73 g/week) and CCPD (1.91 ± 0.63 g/week) patients (Table 2). In CAPD and APD patients, additionally 0.55 ± 0.75 g and 0.67 ± 0.86 g of phosphorus, respectively, were removed by the kidneys within one week (Table 2). In total, the largest amount of phosphorus was removed in CAPD (2.44 ± 0.94 g/week), followed by CCPD (2.02 ± 0.85 g/week) and APD (2.01 ± 1.00 g/week), (Table 2).

### Analysis of correlations

**Figure 1:**
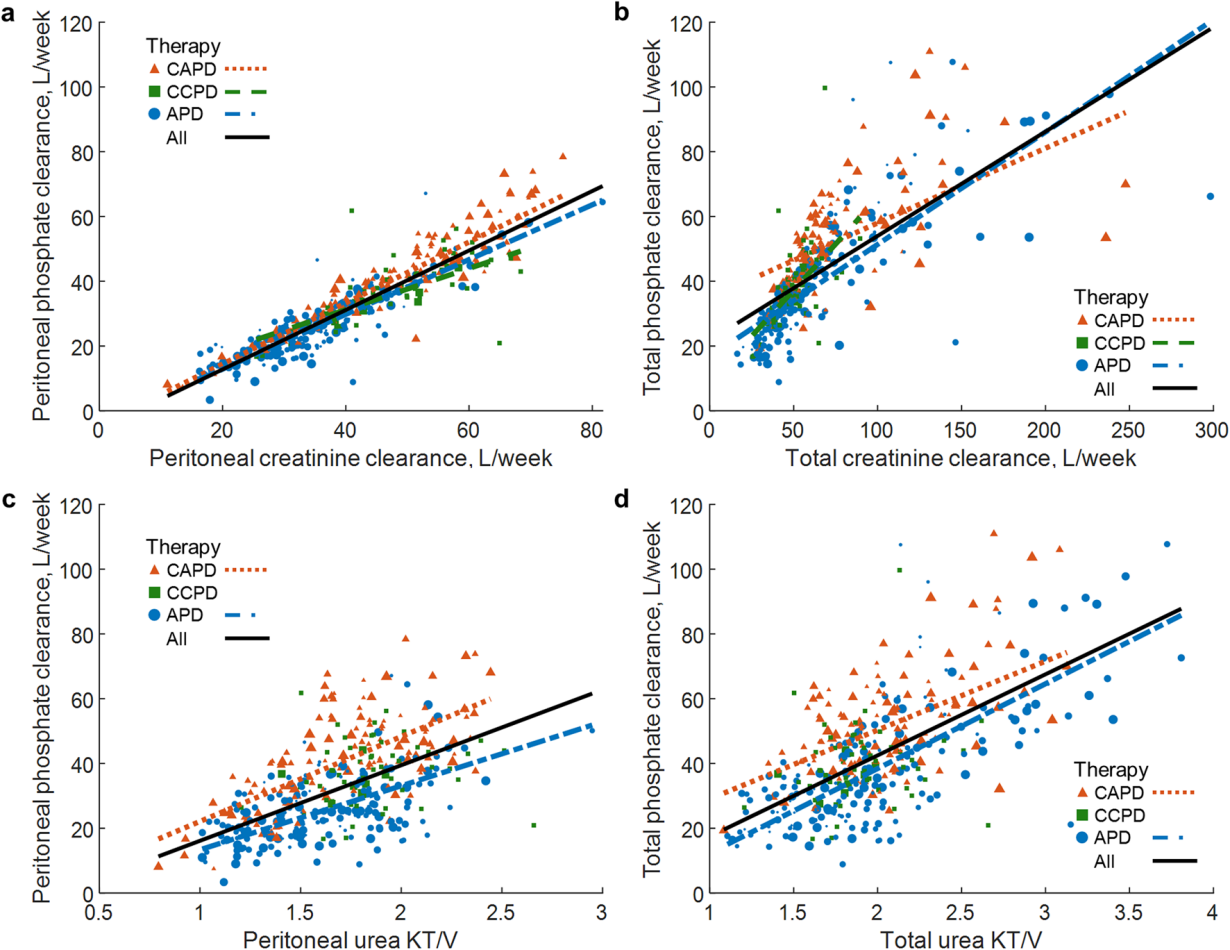
Phosphate clearance versus creatinine clearance and urea KT/V. Peritoneal (**a**) and total (**b**) phosphate clearances versus peritoneal and total creatinine clearances, respectively, as well as peritoneal (**c**) and total (**d**) phosphate clearances versus peritoneal and total urea KT/V, respectively, for continuous ambulatory (CAPD), continuous cyclic (CCPD), automated peritoneal dialysis (APD) and jointly for all therapies. Shown are only regression lines at *p* value < 0.05 (compare Table 3 and Supplementary Table S1). Equations of regression lines for (**a**) CAPD: y = 0.94x − 4.38, CCPD: y = 0.64x + 5.78, APD: y = 0.85x − 4.04, All: y = 0.92x − 5.57; (**b**) CAPD: y = 0.23x + 34.90, CCPD: y = 0.57x + 8.84, APD: y = 0.35x + 16.69, All: y = 0.32x + 21.77; (**c**) CAPD: y = 26.19x − 4.07, APD: y = 19.75x − 6.38, All: y = 23.33x − 7.19 and (**d)** CAPD: y = 21.26x + 7.80, APD: y = 26.16x − 13.87, All: y = 25.03x − 7.54. Size of points reflects the measurement´s weight.

Weekly peritoneal phosphate clearance correlated with peritoneal creatinine clearance (*p* < 0.001) in CAPD (rho = 0.94), CCPD (rho = 0.77) and APD (rho = 0.88) and also when all therapies were pooled (rho = 0.93), (Table 3, Fig. 1a). Similarly, the total phosphate clearance correlated with total creatinine clearance (Supplementary Table S1 and Fig. 1b). Peritoneal phosphate clearance correlated also with creatinine PET D/P in each therapy and for pooled data, but the correlations were weaker (0.29 < rho < 0.51, *p* < 0.01), (Table 3).

**Table 3:** Weighted correlation (Spearman rho) between peritoneal phosphate clearance versus other parameters of patient and therapy for continuous ambulatory (CAPD), continuous cyclic (CCPD), automated peritoneal dialysis (APD) and all pooled therapies.

Weighted Spearman rho	Peritoneal phosphate clearance			
	CAPD	CCPD	APD	All
Age	− 0.14	0.09	0.19**	− 0.08
Weight	− 0.47***	0.51***	− 0.27***	− 0.19***
Height	− 0.24**	0.05	− 0.10	− 0.07
Body mass index	− 0.42***	0.42***	− 0.21**	− 0.15**
Body surface area	− 0.44***	0.50***	− 0.26***	− 0.18***
Total body water	− 0.25**	0.45***	− 0.17*	− 0.07
Serum creatinine	0.08	− 0.04	− 0.12	0.01
Serum urea	0.12	0.12	− 0.01	0.07
Serum phosphorus	− 0.19*	− 0.39**	− 0.12	− 0.27***
Serum glucose	− 0.07	0.07	0.24**	0.10
Serum albumin	− 0.25**	− 0.18	− 0.31***	− 0.14**
Dialysis time	0.40***	0.42***	0.05	0.56***
Cycle no	0.52***	0.29*	0.03	− 0.14**
Infused volume	0.36***	0.45***	0.18*	− 0.03
Drained volume	0.36***	0.45***	0.12	0.00
Ultrafiltration	0.13	0.11	− 0.01	0.16**
Urine volume	− 0.38***	− 0.13	− 0.31***	− 0.32***
Peritoneal urea KT/V	0.70***	0.17	0.62***	0.60***
Renal urea KT/V	− 0.38***	− 0.14	− 0.29***	− 0.32***
Total urea KT/V	0.15	0.10	0.15*	0.14**
Peritoneal creatinine clearance	0.94***	0.77***	0.88***	0.93***
Renal creatinine clearance	− 0.37***	− 0.14	− 0.31***	− 0.32***
Total creatinine clearance	0.11	0.59***	0.09	0.18***
Creatinine PET D/P	0.29**	0.36**	0.51***	0.34***
Renal phosphate clearance	− 0.35***	− 0.14	− 0.26***	− 0.31***
Total phosphate clearance	0.57***	0.95***	0.40***	0.50***
Peritoneal phosphorus removal	0.67***	0.64***	0.69***	0.75***
Renal phosphorus removal	− 0.39***	− 0.14	− 0.29***	− 0.34***
Total phosphorus removal	0.15	0.61***	0.23**	0.24***

‘***’, ‘**’ and ‘*’ denote *p* value < 0.001, < 0.01 and < 0.05, respectively.

In CAPD and APD patients, the peritoneal and total phosphate clearances correlated positively with peritoneal and total urea KT/V, respectively (Table 3 and Supplementary Table S1, Fig. 1c,d). We did not find such dependencies between phosphate clearance and urea KT/V in CCPD patients (Table 3, Supplementary Table S1).

**Figure 2:**
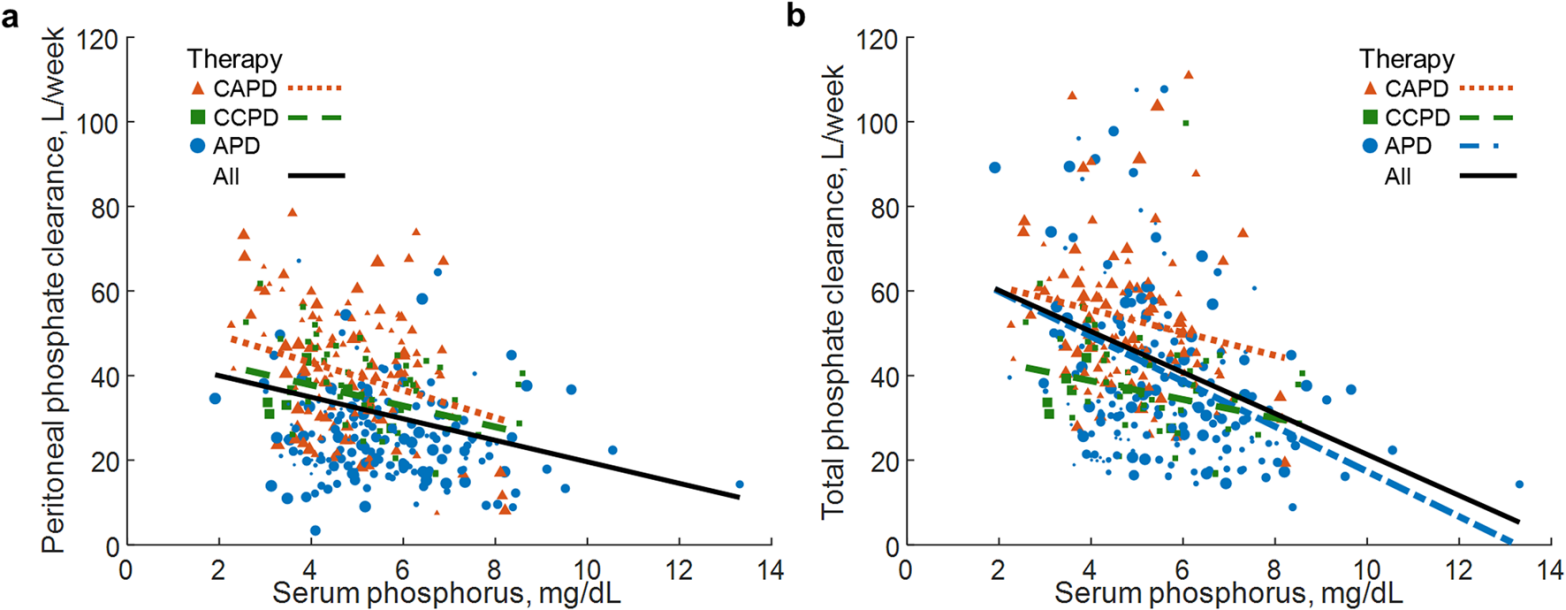
Phosphate clearance versus serum level. Peritoneal (**a**) and total (**b**) phosphate clearances versus serum phosphorus, for continuous ambulatory (CAPD), continuous cyclic (CCPD), automated peritoneal dialysis (APD) and jointly for all therapies. Shown are only regression lines at *p* value < 0.05 (compare Table 3 and Supplementary Table S1). Equations of regression lines for (**a**) CAPD: y =  − 3.25x + 56.05, CCPD: y =  − 2.48x + 47.68, All: y =  − 2.55x + 45.07 and (**b**) CAPD: y =  − 2.70x + 66.40, CCPD: y =  − 2.20x + 47.58, APD: y =  − 5.32x + 70.56, All: y =  − 4.85x + 69.82. Size of points reflects the measurement´s weight.

Higher phosphate clearance was associated with lower serum phosphorus (Fig. 2a,b). There was a negative correlation of peritoneal phosphate clearance with serum phosphorus in CAPD and CCPD patients (Table 3) and negative association of total phosphate clearance with serum phosphorus in each of the studied therapies (Supplementary Table S1). Resulting from the definitions, Eqs. (2) and (3), peritoneal and total phosphorus removals (in g/week) were higher with higher peritoneal and total phosphorus clearances, respectively (Table 3, Supplementary Table S1).

**Figure 3:**
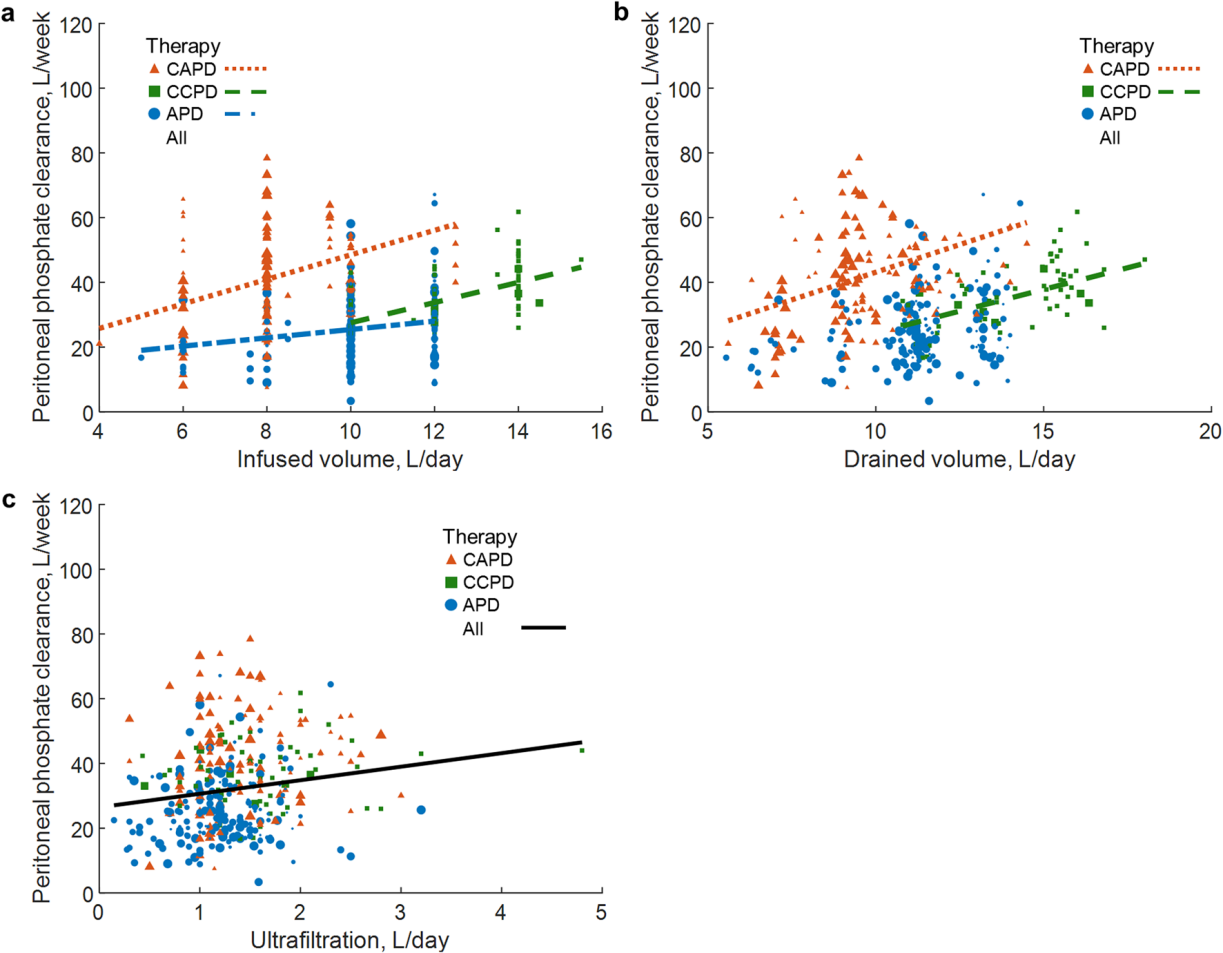
Peritoneal phosphate clearance versus volumes of infusion, drainage and ultrafiltration. Peritoneal phosphate clearance versus infused volume (**a**), drained volume (**b**) and ultrafiltration (**c**), for continuous ambulatory (CAPD), continuous cyclic (CCPD), automated peritoneal dialysis (APD) and jointly for all therapies. Shown are only regression lines at *p* value < 0.05 (compare Table 3). Equations of regression lines for (**a**) CAPD: y = 3.79x + 10.63, CCPD: y = 3.12x − 3.70, APD: y = 1.29x + 12.59; (**b**) CAPD: y = 3.41x + 9.04, CCPD: y = 2.68x − 2.33 and (**c**) All: y = 4.18x + 26.47. Size of points reflects the measurements´ weight.

Peritoneal phosphate clearance correlated positively with dialysis time (rho = 0.56, *p* < 0.001), (Table 3). In 6 (5%) and 17 (28%) cases in CAPD and CCPD, respectively, dialysis time was shorter than 24 h and associated with lower peritoneal phosphate clearance. Higher infused volume in all investigated therapies, and higher drainage volume in CAPD and CCPD, were associated with higher peritoneal phosphate clearance (Table 3, Fig. 3a,b).

### Multivariate regression analysis

In multivariate analysis of predictors influencing phosphate clearance, removal and serum phosphorus, the obtained values of R^2^ showed that phosphate clearance could be predicted quite well (0.65 < R^2^ < 0.74), while phosphorus removal (in g/week) was considerably less predictable (0.31 < R^2^ < 0.46) and serum phosphate level was difficult to predict (R^2^ = 0.28), (Table 4; see also Supplementary Table S2). Multivariate analyses were performed separately in anuric and non-anuric patients (characterized in Supplementary Table S3) and showed that in anuric patients, peritoneal creatinine clearance was the best predictor of peritoneal phosphate clearance (R^2^ = 0.74), (Table 4); in anuric patients with 1 L/week increase of creatinine clearance, the model predicts 0.80 L/week increase of peritoneal phosphate clearance (Table 4). Total phosphate clearance in non-anuric patients was best predicted by renal urea KT/V and peritoneal creatinine clearance (Table 4). Peritoneal phosphorus removal (in g/week) depended on therapy and was positively associated with serum phosphorus and volume of infused dialysis fluid (Table 4). Total phosphorus removal depended on urine volume, total body water and serum phosphorus (Table 4). The increase of phosphate clearance was associated with a decrease of serum phosphorus (Table 4).

**Table 4:** Multivariate regression models of factors predicting peritoneal and total phosphate clearances, removal and serum phosphorus, in anuric and non-anuric patients.

Anuric		Non-anuric	
***Peritoneal phosphate clearance***		***Total phosphate clearance***	
**R**^**2**^** = 0.74**	**B**	**R**^**2**^** = 0.65**	**B**
Intercept	− 0.92	Intercept	− 3.06
Peritoneal creatinine clearance	0.80	Renal urea KT/V	27.38
		Peritoneal creatinine clearance	0.93
***Peritoneal phosphorus removal***		***Total phosphorus removal***	
**R**^**2**^** = 0.46**	**B**	**R**^**2**^** = 0.31**	**B**
Intercept for CAPD	− 0.56	Intercept	− 0.80
Intercept for CCPD	− 0.83	Urine volume	0.0008
Intercept for APD	− 1.16	Total body water	0.04
Serum phosphorus	0.26	Serum phosphorus	0.25
Infused volume	0.16		
***Serum phosphorus***		***Serum phosphorus***	
**R**^**2**^** = 0.28**	**B**	**R**^**2**^** = 0.28**	**B**
Intercept	5.35	Intercept	4.19
Serum urea	0.05	Serum urea	0.04
Peritoneal phosphate clearance	− 0.06	Total phosphate clearance	− 0.02

## Discussion

Our study shows relatively large differences in terms of phosphate removal, clearance and serum levels between patients treated with three different PD modalities: CAPD, CCPD and APD. Whereas the observed differences to a large extent reflect inherent differences between PD modalities as regards number, frequency and volume of dialysis fluid exchanges, as well as differences in patient characteristics, other factors likely play an even more important role. Thus, while dialysis treatment may facilitate reaching a ‘normal’ phosphate level (advocated by current international guidelines without specifying its value^9^), this level depends on many factors other than dialysis such as the interplay between intestinal absorption, exchange with bone, shifts between intracellular and extracellular spaces, and renal excretion of phosphate^10^. Additionally, the resulting mass balance of phosphorus is influenced by several active processes mediated by hormonal regulation by parathyroid hormone, fibroblast-growth-factor-23 and vitamin D^10^. A significant decrease of glomerular filtration rate disrupts phosphate homeostasis and leads to phosphate retention; nevertheless, while in our study the total removal of phosphorus was significantly lower in anuric than in non-anuric patients, their serum phosphorus levels were in fact similar (Supplementary Table S3). Considering that hyperphosphatemia, a late marker of phosphate overload in CKD patients^1^, correlates with poor clinical outcomes^11^, prevention of hyperphosphatemia in dialysis patients is a key priority. Whereas non-dialytic measures such as reduction of phosphate intake, treatment of renal osteodystrophy and use of phosphate binders to reduce absorption of dietary phosphate in gut are essential to prevent hyperphosphatemia^2,6,12^, our study shows that removal of phosphate by dialysis may play a significant role.

In our study, the total weekly removal of phosphorus was larger in patients treated with CAPD than in those treated with CCPD and APD (Table 2), and serum phosphorus was significantly lower in CAPD than in APD patients (Table 1). The dialytic removal of phosphorus was also the subject of other studies but there are a few studies that compare different dialysis modalities^7,13–17^. Typically, hemodialysis was reported to be more effective than PD in terms of phosphorus removal; however, this depends on the subtype of hemodialysis and peritoneal dialysis^7,13–17^. Similar values and patterns of phosphate clearance as in our study were reported in the study by Courivaud and Davenport^18^, 41.4, 33.4 and 16.7 L/week, while we found 41.50, 36.68 and 25.42 L/week (Table 2) in CAPD, CCPD and APD treatments, respectively, suggesting, by both studies better efficiency of CAPD in terms of phosphate elimination. However, whether this difference may have an impact on clinical outcomes is not known. The majority of available comparative analysis have not reported any differences in mortality between CAPD and APD patients^19,20^. Survival analyses that compare subclasses of normo- versus hyper-phosphatemic or high versus low phosphate clearance throughout different PD modalities would possibly reveal whether such differences influence death risk in PD patients.

Clearance, Eqs. (1)–(3), defined as the mass of solute removed from patient body over solute concentration in serum divided by time, during which the mass was removed, is analogous to *equivalent continuous clearance* (ECC), an index that describes dialysis adequacy^21–24^. In contrast to KT/V, ECC does not require the identification of the space, in which the solute is distributed within the body. For urea KT/V, Eqs. (4)–(5), it is assumed that urea is distributed in *total body water*, whereas body distribution of phosphate is not easily identifiable and therefore KT/V is useless in the assessment of dialysis dose in terms of phosphate removal. ECC can be used to compare different therapies, hemodialysis and peritoneal dialysis, or different modalities and schedules, and is applicable to different solutes as urea, creatinine, calcium and phosphate^21–23,25^. Phosphate ECC, normalized as in Eqs. (1)–(2) by 1.73/BSA, was found to be higher in 25 anuric hemodialysis patients than in PD patients analyzed in the current study: 67.54 ± 12.10 L/week^22^ versus 43.44 ± 18.64 L/week (Table 2), respectively. Studies associating phosphate ECC with patient survival on PD and hemodialysis are warranted but for now such analyses have not been conducted. A higher phosphorus level was associated with higher mortality in CKD patients^1,3–5,26^, whereas higher urea KT/V did not associate with patient survival^27^.

In the present study, the correlation between peritoneal phosphate and creatinine clearances was very strong (Table 3, Fig. 1a), also when anuric and non-anuric patients were analyzed separately in multivariate regression model (Table 4). The correlation between peritoneal phosphate clearance and urea KT/V was weaker but still significant in CAPD and APD (Table 3, Fig. 1c). Interestingly, these findings are not fully consistent with the results obtained in hemodialysis patients in whom phosphate clearance (i.e., ECC related to average serum concentration) did not correlate with urea clearance (rho = 0.36, *p* = 0.078) and phosphate ECC correlated less well with creatinine ECC (rho = 0.51, *p* = 0.011)^22^. This means that phosphate clearance can be accurately predicted using creatinine as a proxy marker in PD, whereas in hemodialysis the association between phosphate and creatinine clearances is not convertible^22^. In contrast to the quasi-continuous PD treatment, the hemodialysis procedure represents a substantial disturbance that induces reactions in mineral homeostasis including phosphate inflow to extracellular space and hormonal interactions^28,29^. A ‘passive solute’ such as creatinine does not mimic phosphate behavior when body conditions are rapidly changing^30^.

Higher peritoneal phosphate clearance was found in patients with higher infused volume of dialysis fluid in all investigated therapies (Table 3, Fig. 3a) and peritoneal phosphorus removal was positively associated with infused volume in multivariate model (Table 4), suggesting the influential role of the infused volume in phosphate clearance and removal. On the other hand, phosphate clearance did not correlate with ultrafiltration as peritoneal elimination of phosphate is mainly by diffusion and not by convection (Table 3, Fig. 3c).

The real-world clinical material analyzed in this study was gathered as part of the routine clinical evaluation, not rigorously planned, and hence the statistical methods had to be chosen accordingly. Because of an uneven number of measurements (from 1 to 8) in patients, we could not treat them equally as independent observations. Thus, the relationship between two variables was tested using *weighted* Spearman correlation, in which the sum of weights was 1 in each patient and therefore each patient contributed equally to the results. In multivariate regression, we used the mixed-effects methodology, in which patient was treated as random effect and other variables were set as fixed effects. In other words, we removed influence of different numbers of measurements, treating each patient uniformly, but without losing information from any measurement^31^. Analyzing the three modalities of PD (CAPD, CCPD and APD), the magnitude and direction of correlations of some variables differed between the different therapies (compare Supplementary Figs. S1 and S2). Peritoneal phosphate clearance correlated positively with body mass index and body surface area in CCPD, but negatively in CAPD, APD and for pooled data (Supplementary Figs. S1a, S2a). Similar discrepancies were observed also for other variables, e.g., peritoneal phosphate clearance correlated with ultrafiltration only for pooled data but not in CAPD, CCPD and APD therapies separately (Table 3, Fig. 3c). Therefore, drawing general conclusions regarding correlations between variables in PD, based on one PD modality only, or using pooled data, is not justified until we examine all configurations.

In summary, phosphate clearance (in L/week), phosphorus removal (in g/week) and serum phosphorus differed between the three investigated peritoneal dialysis therapies, i.e., CAPD, CCPD and APD. CAPD seemed to be more effectual strategy as compared to APD. However, it is important to note that the characteristics of patients in the individual therapies differed, perhaps as a consequence of selection biases with subgroups of patients being directed to what appeared to be the most appropriate therapies for them. We agree with Trinh et al. that “modalities are as different as are the patients who choose them”^32^. Consequently, the differences in phosphate removal and serum phosphate between the different investigated PD modalities reported in our study cannot be used to conclusively determine which dialysis method is better or worse. Nevertheless, a better understanding of inherent differences between PD modalities may provide some guidance.

Considering previously reported associations of phosphate control with clinical outcomes, it is tempting to state that phosphorus removal, clearance and serum levels—besides urea KT/V, creatinine clearance and ultrafiltration—should be taken into account in the assessment of PD adequacy and during prescription of optimal therapy. However, studies on outcomes in relation to phosphorus removal—which are currently lacking—are needed for a patient-oriented approach that would allow clinicians to select the best PD modality for specific patients.

## Supplementary information

Supplementary file 1.
