# Supplementary material for: Phosphate clearance in peritoneal dialysis
Source: Sci Rep. 2020 Oct 15;10:17504. doi: 10.1038/s41598-020-74412-2 (PMC7566511; doi:10.1038/s41598-020-74412-2)
Supplement: Supplementary file 1 [file 41598_2020_74412_MOESM1_ESM.pdf]

Supplementary Information

to **“Phosphate clearance in peritoneal dialysis”**

by Malgorzata Debowska, Rafael Gomez, Joyce Pinto, Jacek Waniewski and Bengt Lindholm

Supplementary Table S1

Weighted correlation (Spearman rho) between total phosphate clearance vs. other parameters of patient and therapy for continuous ambulatory (CAPD), continuous cyclic (CCPD) and automated peritoneal dialysis (APD) and for all pooled therapies.

	Total phosphate clearance			
**Weighted Spearman rho**	**CAPD**	**CCPD**	**APD**	**All**
Age	0.08	0.16	0.17*	0.09
Weight	-0.16	0.48***	0.09	0.04
Height	-0.05	0.06	0.00	-0.05
Body mass index	-0.17	0.39**	0.09	0.06
Body surface area	-0.16	0.48***	0.05	0.00
Total body water	0.01	0.45***	0.08	0.04
Serum creatinine	-0.37***	-0.10	-0.60***	-0.48***
Serum urea	0.11	0.13	-0.07	0.04
Serum phosphorus	-0.22*	-0.40**	-0.42***	-0.39***
Serum glucose	-0.01	0.13	0.18*	0.09
Serum albumin	-0.14	-0.23	0.07	-0.02
Dialysis time	0.09	0.45***	-0.14	0.26***
Cycle no	0.21*	0.22	-0.08	-0.28***
Infused volume	-0.02	0.41**	-0.26***	-0.33***
Drainage volume	0.02	0.39**	-0.29***	-0.33***
Ultrafiltration	0.04	0.06	-0.18*	-0.05
Urine volume	0.33***	0.14	0.61***	0.50***
Peritoneal urea KT/V	0.13	0.15	0.12	0.09
Renal urea KT/V	0.37***	0.14	0.67***	0.53***
Total urea KT/V	0.47***	0.19	0.76***	0.65***
Peritoneal creatinine clearance	0.50***	0.73***	0.25***	0.37***
Renal creatinine clearance	0.39***	0.13	0.65***	0.53***
Total creatinine clearance	0.73***	0.70***	0.86***	0.85***
Creatinine PET D/P	0.32***	0.39**	0.13	0.14**
Peritoneal phosphate clearance	0.57***	0.95***	0.40***	0.50***
Renal phosphate clearance	0.44***	0.15	0.71***	0.56***
Peritoneal phosphorus removal	0.35***	0.60***	0.10	0.27***
Renal phosphorus removal	0.38***	0.14	0.67***	0.52***
Total phosphorus removal	0.56***	0.63***	0.76***	0.69***

'***', '**' and '*' denote p-value < 0.001, < 0.01, < 0.05, respectively.

Supplementary Table S2

Weighted correlation (Spearman rho) between serum phosphorus vs. other parameters of patient and therapy for continuous ambulatory (CAPD), continuous cyclic (CCPD), automated peritoneal dialysis (APD) and for all pooled therapies.

	Serum phosphorus			
**Weighted Spearman rho**	**CAPD**	**CCPD**	**APD**	**All**
Age	-0.01	-0.27*	-0.34***	-0.15**
Weight	0.15	-0.43***	-0.07	-0.02
Height	0.04	0.01	0.04	0.05
Body mass index	0.21*	-0.40**	-0.09	-0.04
Body surface area	0.11	-0.36**	-0.03	0.01
Total body water	0.14	-0.29*	-0.02	0.03
Serum creatinine	0.33***	0.28*	0.58***	0.39***
Serum urea	0.40***	0.33**	0.38***	0.37***
Serum glucose	-0.02	-0.08	-0.09	-0.05
Serum albumin	0.13	0.13	0.10	0.02
Dialysis time	-0.10	-0.38**	0.02	-0.24***
Cycle no	-0.10	-0.32*	0.02	0.07
Infused volume	-0.03	-0.26*	0.11	0.10*
Drainage volume	-0.06	-0.16	0.16*	0.10
Ultrafiltration	-0.06	0.08	0.21**	0.00
Urine volume	0.11	-0.01	-0.24***	-0.06
Peritoneal urea KT/V	-0.24**	-0.12	0.02	-0.12*
Renal urea KT/V	0.02	-0.04	-0.33***	-0.13*
Total urea KT/V	-0.33***	-0.14	-0.35***	-0.32***
Peritoneal creatinine clearance	-0.22*	-0.49***	-0.13	-0.28***
Renal creatinine clearance	0.00	-0.04	-0.34***	-0.14**
Total creatinine clearance	-0.23*	-0.49***	-0.46***	-0.40***
Creatinine PET D/P	0.11	-0.20	-0.29***	-0.12*
Peritoneal phosphate clearance	-0.19*	-0.39**	-0.12	-0.27***
Renal phosphate clearance	0.10	-0.05	-0.31***	-0.10
Total phosphate clearance	-0.22*	-0.40**	-0.42***	-0.39***
Peritoneal phosphorus removal	0.43***	0.36**	0.52***	0.34***
Renal phosphorus removal	0.20*	-0.02	-0.20**	0.00
Total phosphorus removal	0.56***	0.35**	0.19*	0.29***

'***', '**' and '*' denote p-value < 0.001, < 0.01 and < 0.05, respectively.

Supplementary Table S3
Patient and therapy characteristics in anuric vs. non-anuric group.

	**Anuric**	**Non-anuric**
Number of patients[(a)]	64 (41%)	92 (59%)
Number of measurements	172 (47%)	196 (53%)
Transport type, 1/2/3/4[(b)]	16%/32%/38%/13%	20%/33%/34%/10%
Creatinine PET D/P	0.64 ± 0.12	0.62 ± 0.13
Urine volume, mL/day	0.00 ± 0.00	644.17 ± 458.83***
Gender, male	58%	55%
Age, year	52.07 ± 18.16	54.21 ± 17.00
Weight, kg	65.26 ± 13.03	66.01 ± 14.14
Height, cm	1.64 ± 0.09	1.62 ± 0.09
Body mass index, kg/m^2	24.39 ± 4.59	24.91 ± 4.30
Body surface area, m^2	1.70 ± 0.18	1.70 ± 0.21
Total body water, L	36.19 ± 6.15	35.53 ± 6.21
Serum creatinine, mg/dL	13.41 ± 3.26	10.54 ± 4.60***
Serum urea, mg/dL	46.19 ± 13.74	46.21 ± 13.04
Serum phosphorus, mg/dL	5.43 ± 1.65	5.11 ± 1.32
Serum glucose, mg/dL	126.74 ± 71.11	116.29 ± 55.38
Serum albumin, mg/dL	3.73 ± 0.61	3.74 ± 0.50
Dialysis time h/day	16.95 ± 6.70	15.73 ± 6.84*
Number of cycles	5.02 ± 0.83	4.40 ± 0.83***
Infused volume, L/day	11.10 ± 1.82	8.93 ± 1.89***
Drainage volume, L/day	12.51 ± 1.97	10.19 ± 2.00***
Ultrafiltration, L/day	1.41 ± 0.54	1.26 ± 0.51**
Peritoneal urea KTV	1.79 ± 0.30	1.58 ± 0.35***
Renal urea KTV	0.00 ± 0.00	0.62 ± 0.50***
Total urea KTV	1.79 ± 0.30	2.19 ± 0.51***
Peritoneal creatinine clearance, L/week	44.99 ± 11.92	37.71 ± 14.09***
Renal creatinine clearance, L/week	0.00 ± 0.00	44.68 ± 44.34***
Total creatinine clearance, L/week	44.99 ± 11.92	82.39 ± 42.57***
Peritoneal phosphate clearance, L/week	35.31 ± 12.11	30.09 ± 14.74***
Renal phosphate clearance, L/week	0.00 ± 0.00	20.47 ± 17.30***
Total phosphate clearance, L/week	35.31 ± 12.11	50.56 ± 20.40***
Peritoneal phosphorus removal, g/week	1.81 ± 0.70	1.44 ± 0.68***
Renal phosphorus removal, g/week	0.00 ± 0.00	1.01 ± 0.85***
Total phosphorus removal, g/week	1.81 ± 0.70	2.45 ± 1.08***

[(a)] 2 patients switched from non-anuric to anuric.
[(b)] 1, 2, 3, 4 denote slow, slow average, fast average and fast transport types, respectively; in 1% of measurements in anuric and 3% in non-anuric transport type was not evaluated.
'*', '**' and '***' denote p-value < 0.05, < 0.01 and 0.001 vs. anuric, respectively.

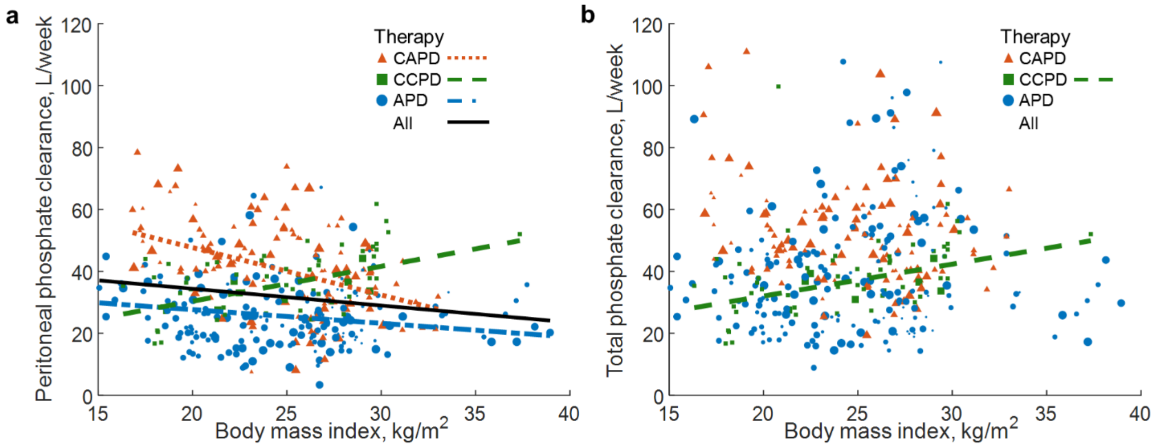

Supplementary Fig. S1

Phosphate clearance vs. body mass index. Peritoneal (panel a) and total (panel b) phosphate clearances vs. body mass index, for continuous ambulatory (CAPD), continuous cyclic (CCPD), automated peritoneal dialysis (APD) and for all pooled therapies. Shown are only regression lines at p-value < 0.05 (compare Table 3 and Supplementary Table S1). Equations of regression lines for panel a: CAPD: y=-1.53x+78.29, CCPD: y=1.13x+7.83, APD: y=-0.44x+36.50, All: y=-0.54x+45.25 and panel b: CCPD: y=1.03x+11.48. Size of points reflects the measurement´s weight.

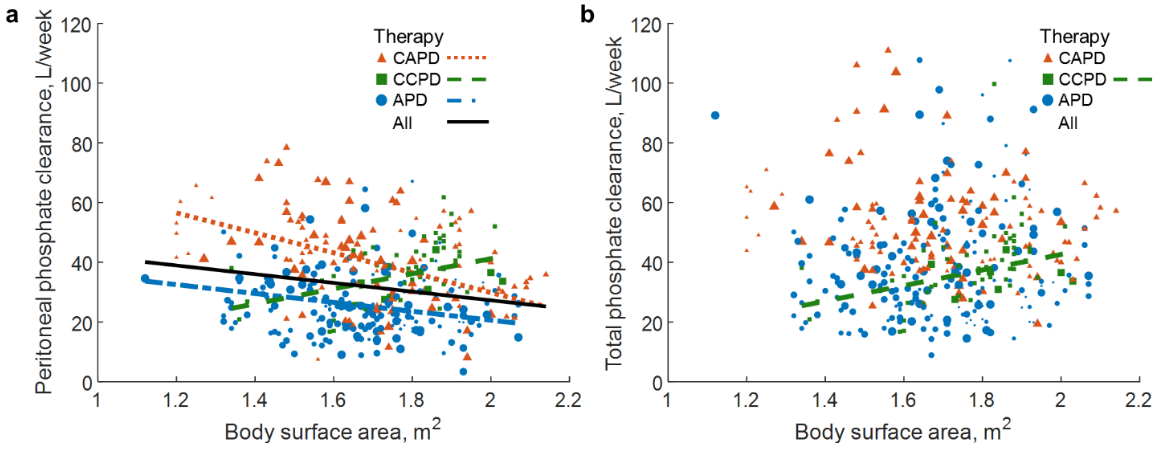

Supplementary Fig. S2

Phosphate clearance vs. body surface area. Peritoneal (panel a) and total (panel b) phosphate clearance vs. body surface area for continuous ambulatory (CAPD), continuous cyclic (CCPD), automated peritoneal dialysis (APD) and for all pooled therapies. Shown are only regression lines at p-value < 0.05 (compare Table 3 and Supplementary Table S1). Equations of regression lines for panel a: CAPD: y=-33.39x+96.61, CCPD: y=25.21x-9.22, APD: y=-14.89x+50.39, All: y=-14.59x+56.44 and panel b: CCPD: y=26.05x-9.47. Size of points reflects the measurement´s weight.
